# Case of Uterine Pregnancy of over Twenty Years Standing

**Published:** 1850-11

**Authors:** William D. Christian

**Affiliations:** Appomattox Co., Virginia


*Case of Uterine Pregnancy of over twenty years standing.* By William D. Christian, M. D., of Appomattox Co., Virginia.—Mrs. T., aged about 40. In the early part of the summer of 1848, Dr. D. and myself were called to visit Mrs. T. We found her suffering with violent pain in the abdomen, attended with obstinate constipation. On making a minute examination of the case, we found a considerable tumor occupying the right iliac and right lumbar regions. Inquiry being made in relation to said tumor, duration, &c., we received from her the following statement: About twenty years ago, and not long after her marriage, she conceived, or felt all the symptoms of a conception, passed through the regular stages of pregnancy, quickening at regular time, &c., and at the end of her full term, nine months, she was taken with ordinary labor pains. In anticipation of a speedy delivery, a midwife was summoned. After remaining in labor for a day or two, (during which time she felt distinctly the motions of the fœtus ) the motions of the fœtus suddenly ceased, and very soon after the labor pains subsided. A distinguished physician of Campbell county, Dr. S., was then called in to see her. The following is an extract from a letter received from him in answer to certain inquiries made by us of him in relation to this case:

"I visited Mrs. T. on the 8th of December, 1827; the old lady in attendance, then supposed her to have been in labor a day or two, and from the history of the case I presumed she was correct; upon examination, I found the *parts* moderately dilated, and in some short time after the examination, there were expelled from the uterus some two or three bunches of hydatids, varying in size from large shot to partridge eggs. After this, she became much relieved, and in an hour or two I left her again in the hands of the midwife, who informed me afterwards that she continued to improve. Early in the year 1828, she removed from the neighborhood, and I saw her no more until the 9th of January, 1833, when, on examination, I found the tumor described by you. I did not renew my visit, nor did I have any conversation with her on this subject, until the 5th of August, 1843, when I was again called to see her, renewed my examination, and then became fully convinced it was a case of extra uterine pregnancy, (having had, in the meantime, a case similar to your description of Mrs. T.'s, excepting that the woman had no children after this conception, nor was there any ossification of the placenta; this case was one of twenty-five years standing.) I made known to Mrs. T., at this time, my opinion of her case, and she requested me to make a post-mortem examination at her death, and 'ascertain all about the matter.' I need not add that I am truly gratified you have done so."

During our attendance on Mrs. T., she exhibited signs of having again conceived. This was her eighth conception after the appearance of the tumor. She was then the mother of seven living children. On the 9th of December, 1848, we were summoned to attend her in labor, with this eighth fœtus; the labor was an ordinary one, the tumor not at all interfering; she did remarkably well, getting up unusually soon. Early in January she left the neighborhood, and was taken ill soon after her arrival at her new home, of which sickness she soon after died. We were requested by the physician who had charge of the case in her last illness, to aid him in making a post-mortem examination. On opening the abdomen, we found a compact tumor (resembling in shape and appearance an ostrich egg, though much larger), adhering closely to the walls of the abdomen anteriorly, and completely enveloped in a bony structure, except the upper part, which was the cranium of the fœtus. On cutting through this bony envelope, or sack, we found a perfect child, very much compressed, occupying the smallest possible space, and about the size of a six or seven months fœtus, but as perfect in all respects as a nine months one, and not in the least degree decomposed. The cord was entire, and adhering to the inner part of the sac, which was of a soft bony structure, as before remarked, and evidently the placenta. The edges of this bony placenta were firmly united to the frontal, parietal and occipital bones, thus, with the cranium of the fœtus, forming a complete osseous sac; there were also attachments at the elbows and knees. In this case, nature had protected the mother from danger, by making a covering for the fœtus, of the placenta, and converting this into an osseous structure. I am not aware of any case being reported in which the child was enveloped in a bony sac. Death, in Mrs. T.'s case, was purely mechanical; by getting up too soon after delivery, and while the muscles of the abdomen were relaxed, the tumor, by its gravity, fell into the pelvis, drawing the abdominal parietes after it), and thereby pressing on the large intestine, and stopping the alvine discharges &c., thus causing death. As a matter of course, the left ovary was in a healthy condition, for she could not have conceived after this case of extra uterine conception, had it not been so, unless we suppose the right ovary was healthy, with this unusual condition of things in its region, which was not true in this case, and is not likely to be in any other case like it.—*Medical Ex., and Rec. of Med. Sci.*

